# Chamber oxygen concentration impacts mitochondrial function and hydrogen peroxide appearance in permeabilized human skeletal muscle fibers

**DOI:** 10.1016/j.bbabio.2025.149568

**Published:** 2025-08-11

**Authors:** Bradley A. Ruple, Soung Hun Park, Jesse C. Craig, Matthew T. Lewis, Joel D. Trinity, Russell S. Richardson, Ryan M. Broxterman

**Affiliations:** a Geriatric Research, Education, and Clinical Center, George E. Wahlen VA Medical Center, Salt Lake City, UT, United States of America; b Department of Internal Medicine, Division of Geriatrics, University of Utah, Salt Lake City, UT, United States of America; c Department of Nutrition and Integrative Physiology, University of Utah Salt Lake City, UT, United States of America

**Keywords:** Oxygen availability, Maximal respiration, Reactive oxygen species

## Abstract

Skeletal muscle mitochondrial respiration is commonly assessed ex vivo using permeabilized fibers in media with high oxygen (O_2_) concentrations to ensure that O_2_ availability does not limit respiration. However, high O_2_ concentrations also increase the production of reactive O_2_ species that can negatively affect respiration. In this study, we tested the hypotheses that permeabilized fiber mitochondria in a high, compared to low, O_2_ concentration would (i) not be different at maximal state 3 respiration rate (V_max_), (ii) have lower submaximal respiration rates at submaximal O_2_ concentrations, and (iii) have greater total cumulative hydrogen peroxide (H_2_O_2_) appearance. We continuously monitored mitochondrial state 3 respiration and H_2_O_2_ appearance rates using high-resolution respirometry in permeabilized skeletal muscle fibers (12 untrained participants; 22 ± 4 yrs) with either control (~127 mmHg; CON) or high (~327 mmHg; HIGH) partial pressures of O_2_ (PO_2_). V_max_ was not different between conditions (HIGH: 80.7 ± 16.7 vs. CON: 82.3 ± 18.7 pmol/s/mg, *p* = 0.695). The PO_2_ at 80 % V_max_ (P_80_) was greater in HIGH (73.9 ± 25.5 vs. 28.0 ± 7.1 mmHg, *p* < 0.001) and respiration rates at 5–60 mmHg PO_2_ were lower for HIGH than CON (all *p* < 0.001). Additionally, the total cumulative H_2_O_2_ appearance was greater in HIGH than CON (*n* = 11; 51.5 ± 23.2 vs. 18.3 ± 10.3 pmol/mg, *p* < 0.001), and this difference was directly correlated with the difference in P_80_ (*r* = 0.655, *p* = 0.029). The current findings support that a high O_2_ concentration, by itself, does not appear to affect V_max_ in the permeabilized skeletal muscle fiber preparation, but the corollary increase in H_2_O_2_ exposure may diminish mitochondrial state 3 respiratory function.

## 1. Introduction

Mitochondria are critical organelles responsible for energy production in skeletal muscle through the generation of adenosine triphosphate (ATP) via aerobic respiration (i.e., mitochondrial respiration). Researchers routinely assess mitochondrial respiration using high-resolution respirometry to reveal the effects of diseases [1–3], interventions [4–6], and substrate utilization [7,8] on skeletal muscle mitochondrial bioenergetics. These studies commonly assess mitochondrial function in permeabilized muscle fibers ex vivo by measuring the rate of O_2_ consumption coupled to ATP production (i.e., state 3 respiration), which occurs in the presence of ADP and reflects active, phosphorylating respiration in response to energy demand [9–11]. State 3 respiration rate is dependent on O_2_ as a substrate for cytochrome *c* oxidase (complex IV), and experimental protocols typically utilize relatively high O_2_ concentrations in the respiration chamber to ensure O_2_ availability does not limit respiration [12]. Critically, these high O_2_ concentrations may directly or indirectly impair mitochondrial state 3 respiratory function, though this needs further investigation in permeabilized skeletal muscle fibers.

In skeletal muscle mitochondria, there is a complex relationship between the O_2_ concentration, production of reactive O_2_ species (ROS), and state 3 respiration rate. To elicit the maximal state 3 respiration rate (V_max_), the permeabilized skeletal muscle fiber technique requires relatively high O_2_ concentrations in the respiratory medium to avoid diffusion limitations caused by the distance between mitochondria embedded in the intact myofibrils and the respiration media [9,12]. A previous study found that O_2_ availability limited V_max_ at a chamber partial pressure of O_2_ (PO_2_) of ~36 mmHg but not at ~140 mmHg in permeabilized human skeletal muscle fiber mitochondria [13]. That study also reported a greater mitochondria hydrogen peroxide (H_2_O_2_) appearance for the higher O_2_ concentration condition. Another study observed that mitochondrial H_2_O_2_ appearance was greater with higher O_2_ concentrations in permeabilized mouse skeletal muscle fibers [14]. Critically, the greater ROS production at high O_2_ concentrations may detrimentally affect mitochondria V_max_, submaximal state 3 respiration rate, or uncoupled respiration (i.e., LEAK respiration) [13,15,16], but this has not been elucidated in permeabilized human skeletal muscle fibers.

Therefore, this study investigated the effects of O_2_ availability on mitochondrial state 3 respiration and H_2_O_2_ appearance rates in permeabilized human skeletal muscle fibers. We specifically examined how control and high O_2_ concentrations affect mitochondrial state 3 respiration and H_2_O_2_ appearance rates across a dynamic range of O_2_ availability, starting at V_max_ and continuing until respiration ceased. We tested the hypotheses that permeabilized fiber mitochondria in high, compared to control, would (i) not be different at V_max_, (ii) have lower submaximal respiration rates at submaximal O_2_ concentrations, and (iii) have greater total cumulative H_2_O_2_ appearance.

## 2. Methods

### 2.1. Ethical approval and participants

The Institutional Review Boards at the University of Utah and Salt Lake City Veterans Affairs Medical Center reviewed and approved this study, which conformed to the standards of the *Declaration of Helsinki* except for registration in a database. Participants were informed of the procedures and potential risks of participating in the study, and each participant provided verbal and written consent prior to participating in the study. A group of 12 participants (age: 22 ± 4 years; sex: 6 Female/6 Male; BMI: 24.7 ± 4.5 kg/m^2^) participated in the current study. All participants were free of overt chronic disease and did not engage in frequent exercise. The participants were instructed to fast for at least 4 h and refrain from vigorous exercise for at least 24 h prior to their experimental visit.

### 2.2. Skeletal muscle biopsy

Muscle samples were obtained under sterile conditions from the vastus lateralis, ~15 cm proximal to the knee. Approximately 10min after administering the local anesthetic (lidocaine), we used a sterile scalpel to make a small incision through which we inserted a 5-gauge biopsy needle to ~2cm below the muscle fascia. Immediately following the biopsy, 30–50 mg of the muscle was stored in ice-cold biopsy preservation fluid (BIOPS; 2.77 mM CaK_2_EGTA, 7.23 mM K_2_EGTA, 6.56 mM MgCl_2_, 0.5 mM DTT, 50 mM K-MES, 20 mM imidazole, 20 mM taurine, 5.77 mM Na_2_ATP, and 15 mM phosphocreatine, pH 7.1 at 4 °C) for the subsequent respiratory analyses.

### 2.3. Mitochondrial respiration and H_2_O_2_ production

Adipose and connective tissues were removed from the muscle fibers in 4 °C BIOPS by careful use of fine-tip forceps under a microscope (SZX10; Olympus, Center Valley, PA, USA). The muscle fibers were then permeabilized with Saponin in BIOPS (50 mg/ml) for 30 min. Saponin is a mild detergent that selectively permeabilizes the sarcolemma due to its high cholesterol content, while mitochondrial membranes remain unaffected at this concentration due to their lower cholesterol content [17]. Following permeabilization, the muscle fibers were rinsed twice for 10 min in mitochondrial respiration medium (MIR05; 2.77 mM CaK2EGTA, 7.23 mM K2EGTA, 6.56 mM MgCl_2_, 0.5 mM DTT, 20 mM imidazole, 5.77 mM ATP, 15 mM phosphocreatine, 50 mM K-MES, and 20 mM taurine, pH 7.0).

**Fig. 1.**
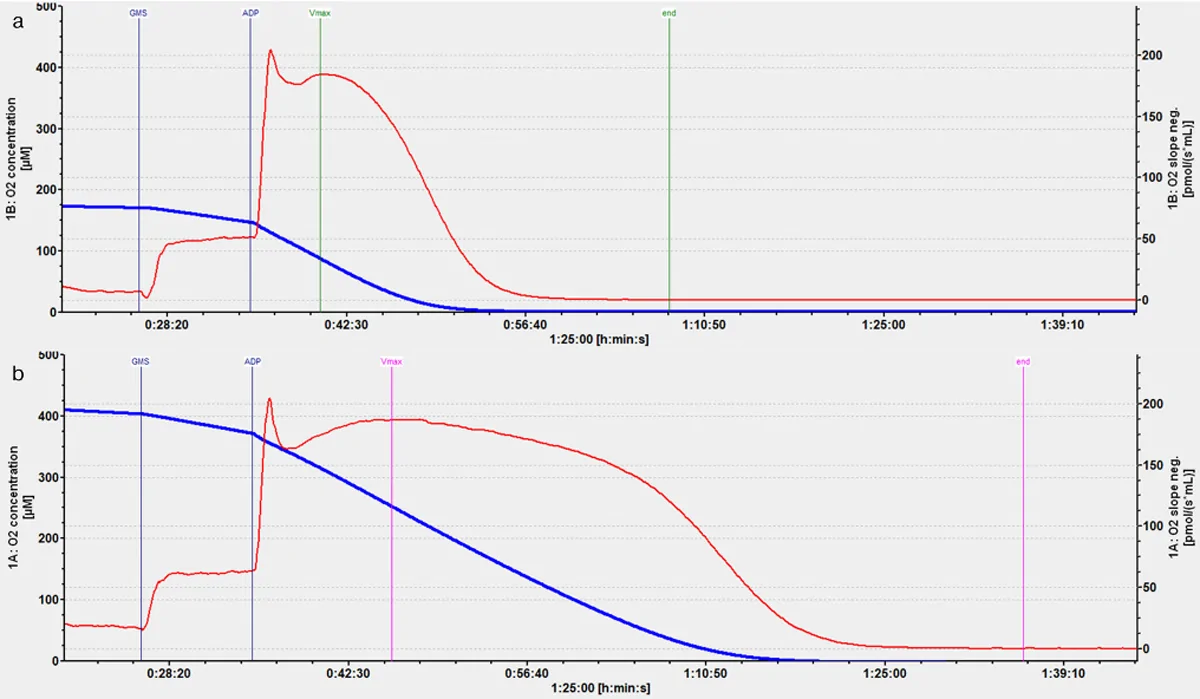
Experimental protocols and Oxygraph-2 k tracings in permeabilized skeletal muscle fibers with control and high initial O_2_ concentrations in a representative sample. Panel a: CON, control initial PO_2_ (≈127 mmHg). Panel b: HIGH, high initial PO_2_ (≈327 mmHg). The blue lines represent the O_2_ concentration, while the red lines represent the O_2_ consumption in the respiration chamber. Substrates glutamate (2 mM), malate (10 mM), and succinate (10 mM) were added at the GMS indicator line to stimulate Complex I + II LEAK respiration. ADP (2.5 mM) was added at the ADP indicator line to stimulate Complex I + II state 3 respiration. The V_max_ indicator line represents where V_max_ was attained. The end indicator line represents where respiration ceased. Data shown are from one participant and are representative of the experimental protocols.

High-resolution respirometry (O2k, OROBOROS Instrument, Innsbruck, Austria) was used for the mitochondrial respiratory analyses, and all measurements were performed once per sample. Fig. 1 presents the experimental protocols and O2k tracings from a representative muscle sample. Briefly, permeabilized muscle fibers were placed in an O2k-chamber containing 2 ml of the MIR05 solution that was continuously stirred and maintained at 37 °C. For the control O_2_ concentration condition (CON), we left the respiration chamber open to room air until the O_2_ concentration in the MIR05 solution attained steady values (mean: 123 mmHg; range: 116–129 mmHg). For the high O_2_ concentration condition (HIGH), we injected 100 % O_2_ gas into the respiration chamber, before fully closing the stopper, to attain hyperoxic values in the MIR05 solution (mean: 327 mmHg; range: 295–425 mmHg). Respiration and H_2_O_2_ appearance rates were measured simultaneously throughout each experimental protocol. In the absence of ADP, mitochondrial Complex I + II LEAK respiration and H_2_O_2_ appearance rates were assessed following the addition of glutamate (2 mM), malate (10 mM), and succinate (10 mM). Next, ADP (2.5 mM) was added to the respiration chamber to assess Complex I + II state 3 respiration and H_2_O_2_ appearance rates. The protocol continued until respiration ceased, defined by a plateau in both O_2_ consumption and chamber O_2_ concentration, indicating that the mitochondria could no longer effectively utilize O_2_.

To measure the H_2_O_2_ and superoxide appearance rate from the mitochondria we added Amplex UltraRed (10 μM), horseradish peroxidase (1 U/ml), and superoxide dismutase (5 U/ml) to the respiration chamber prior to establishing to O_2_ concentration in the MIR05 solution. Catalyzed by the horseradish peroxidase, the Amplex UltraRed reacts with H_2_O_2_ in the MIR05 solution to form resorufin, which was measured using Fluorescence-Sensor Green of the LED2-Module (O2k-Fluo, OROBOROS Instrument, Innsbruck, Austria). The H_2_O_2_ data are presented as appearance, and not production, because the measurement only captures H_2_O_2_ in the solution and does not account for any ROS that is scavenged or remains in the mitochondria. Additionally, any superoxide in the respiration medium is converted to H_2_O_2_ by the added superoxide dismutase, so that the measured H_2_O_2_ values represented both H_2_O_2_ and superoxide appearance from the mitochondria. As H_2_O_2_ and superoxide are rapidly converted to resorufin, their concentrations in the respiration medium do not increase. Before each experiment, we generated a H_2_O_2_ standard curve to convert the fluorescence signal to a H_2_O_2_ appearance value.

### 2.4. Data and statistical analyses

Mitochondrial respiration and H_2_O_2_ appearance rates were corrected for non-mitochondrial background signals, which we measured after respiration ceased. These signals likely reflect non-specific fluorescence or residual chamber activity and therefore were subtracted from all preceding data. The rates were normalized to the dry muscle tissue mass, which was measured after the protocol ended. Mitochondrial respiration rate, chamber O_2_ concentration, and H_2_O_2_ appearance were measured every 2 s throughout each protocol from when respiration initially attained steady values until respiration ceased. We also averaged these data into bins for the range of chamber O_2_ concentration and the range of time that were common to both CON and HIGH. This range of chamber O_2_ concentrations was 0.1–60 mmHg, which was binned as 0.1–4.9, 5.0–10.0, 10.1–20.0, 20.1–30.0, 30.1–40.0, 40.1–50.0, 50.1–60.0 mmHg. The range of time was from 0 to 1100 s, which was separated into 100-s bins.

For both the CON and HIGH conditions, we determined the V_max_, PO_2_ corresponding to 80 % V_max_ (P_80_), respiratory control rate (RCR), peak rate of H_2_O_2_ appearance, and cumulative H_2_O_2_ appearance. The RCR was calculated by state 3/LEAK respiration [18]. We determined V_max_ as the highest respiration rate for each protocol. We determined the peak rate of H_2_O_2_ appearance as the highest rate for each protocol and the cumulative H_2_O_2_ appearance relative to time as the area under the curve above baseline for each protocol. Statistical analyses were performed using GraphPad Prism (Version 10.0.2; San Diego, CA). Two-way (bin x condition) repeated measures ANOVAs and Holm-Šídák post hoc testing were performed for both state 3 respiration rate and H_2_O_2_ appearance. Comparisons between CON and HIGH were made using paired samples *t*-tests for the V_max_, P_80_, RCR, peak rate of H_2_O_2_ appearance, and cumulative H_2_O_2_ appearance. We assessed the associations between select variables using Pearson correlations and we interpreted the strength of the correlation coefficients using previous recommendations [19]. For all statistical analyses, a *p*-value <0.05 was considered significant, and all data are expressed as mean ± standard deviation.

## 3. Results

### 3.1. Mitochondrial respiration in CON and HIGH

**Fig. 2.**
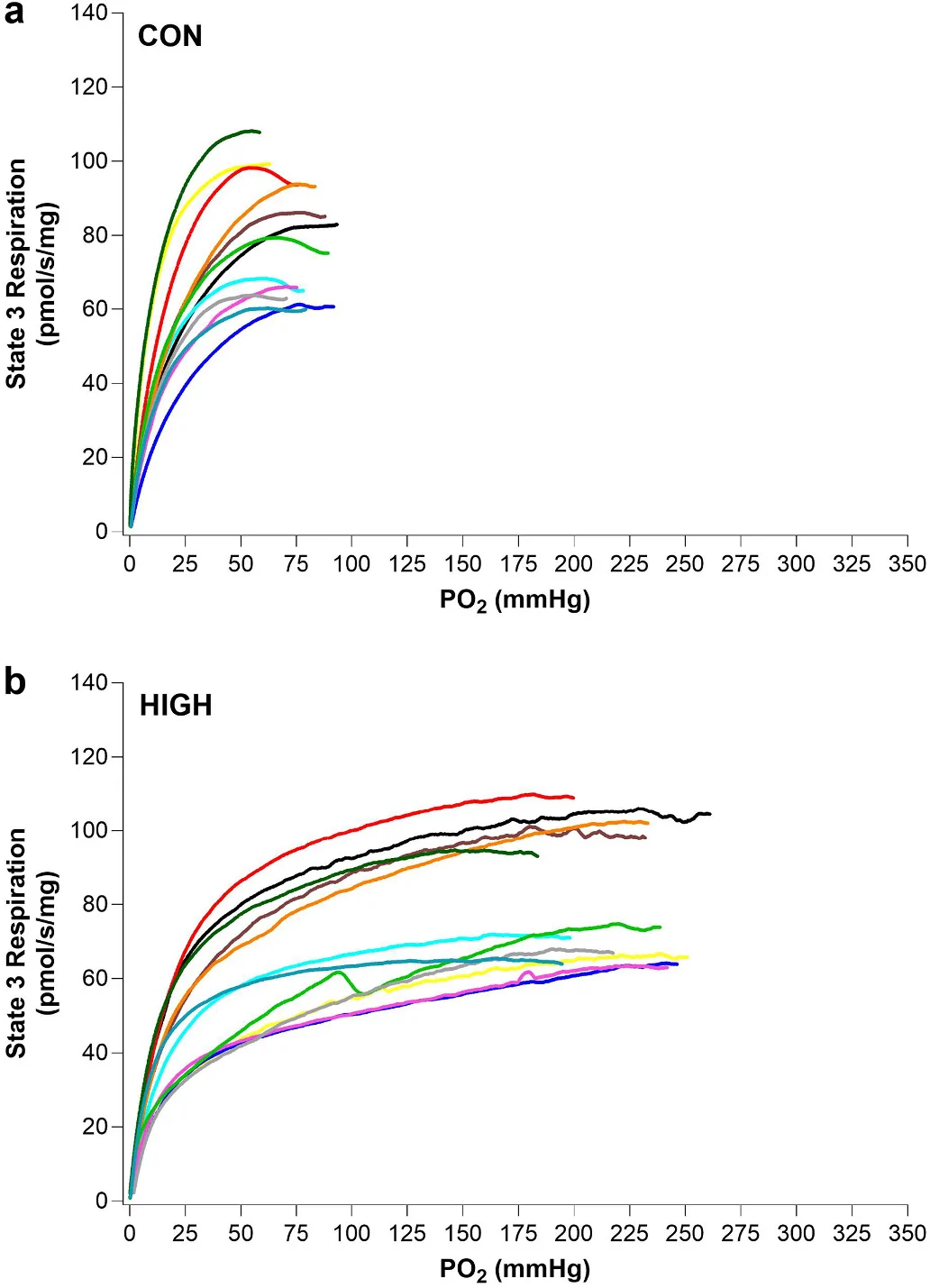
Individual data documenting the effect of falling PO_2_ on mitochondrial respiration rate in permeabilized skeletal muscle fibers with control and high initial O_2_ concentrations. Panel a: CON, control initial PO_2_ (≈127 mmHg). Panel b: HIGH, high initial PO_2_ (≈327 mmHg). Corresponding colors between panels indicate the same participant.

**Fig. 3.**
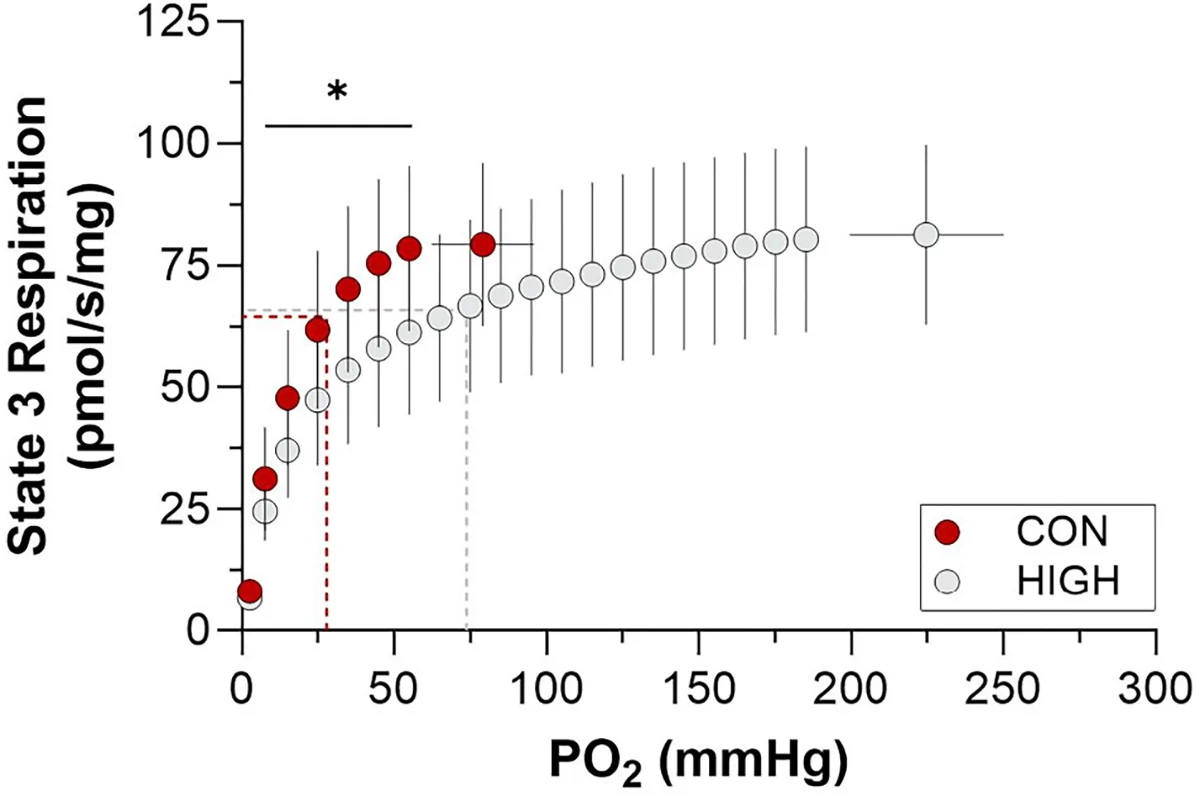
The effect of falling PO_2_ on mitochondrial respiration rate in permeabilized skeletal muscle fibers with control and high initial O_2_ concentrations. CON, control initial PO_2_ (≈127 mmHg); HIGH, high initial PO_2_ (≈327 mmHg). The symbols at the highest PO_2_ for each condition indicate the maximal mitochondrial state 3 respiration rates (V_max_). The dotted lines indicate the PO_2_ at 80 % V_max_ (P_80_). Data are expressed as mean ± SD. *n* = 12 participants. * Significant difference between CON and HIGH at a given PO_2_, *p* < 0.05.

**Fig. 4.**
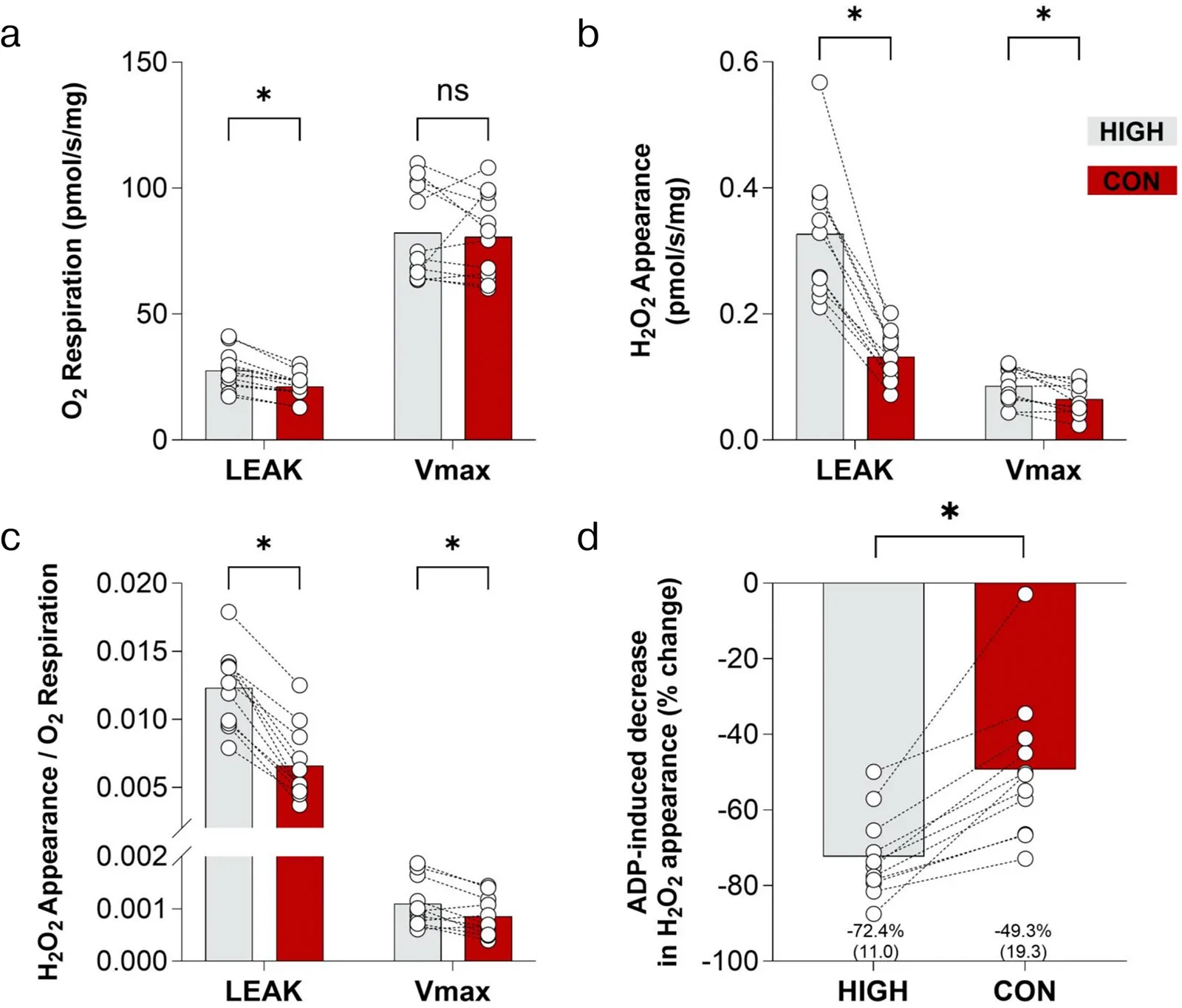
The mitochondrial respiration rate, mitochondrial H_2_O_2_ appearance, and the ADP-induced decrease in H_2_O_2_. CON, control initial PO_2_ (≈127 mmHg); HIGH, high initial PO_2_ (≈327 mmHg). Panel a: Mitochondrial uncoupled respiration rate in the absence of ADP (LEAK) and maximal state 3 respiration rate (V_max_). Panel b: Mitochondrial H_2_O_2_ appearance at LEAK and V_max_. Panel c: H_2_O_2_ appearance normalized to respiration at LEAK and V_max_. Panel d: ADP-induced decrease in H_2_O_2_ appearance from LEAK to V_max_. Data are expressed as mean ± SD. * Significant difference between CON and HIGH, *p* < 0.05.

The individual data for mitochondrial respiration rates as a function of PO_2_ are presented in Fig. 2. While V_max_ was not significantly different between CON and HIGH (*p* = 0.695, Figs. 3 & 4a), LEAK respiration was significantly greater in HIGH (27.6 ± 7.7 pmol/s/mg vs. 21.3 ± 5.1 pmol/s/mg, *p* < 0.001, Fig. 4a). RCR was significantly different between conditions (3.1 ± 0.5 HIGH vs 3.9 ± 0.8 CON, *p* < 0.001). There were significant effects for PO_2_ (*p* < 0.001), condition (*p* = 0.008), and their interaction (*p* < 0.001) for state 3 respiration rate. Specifically, the state 3 respiration rate was significantly lower in HIGH than CON for the 7.5–55 mmHg PO_2_ bins (*p* < 0.001, Fig. 3).

**Fig. 5.**
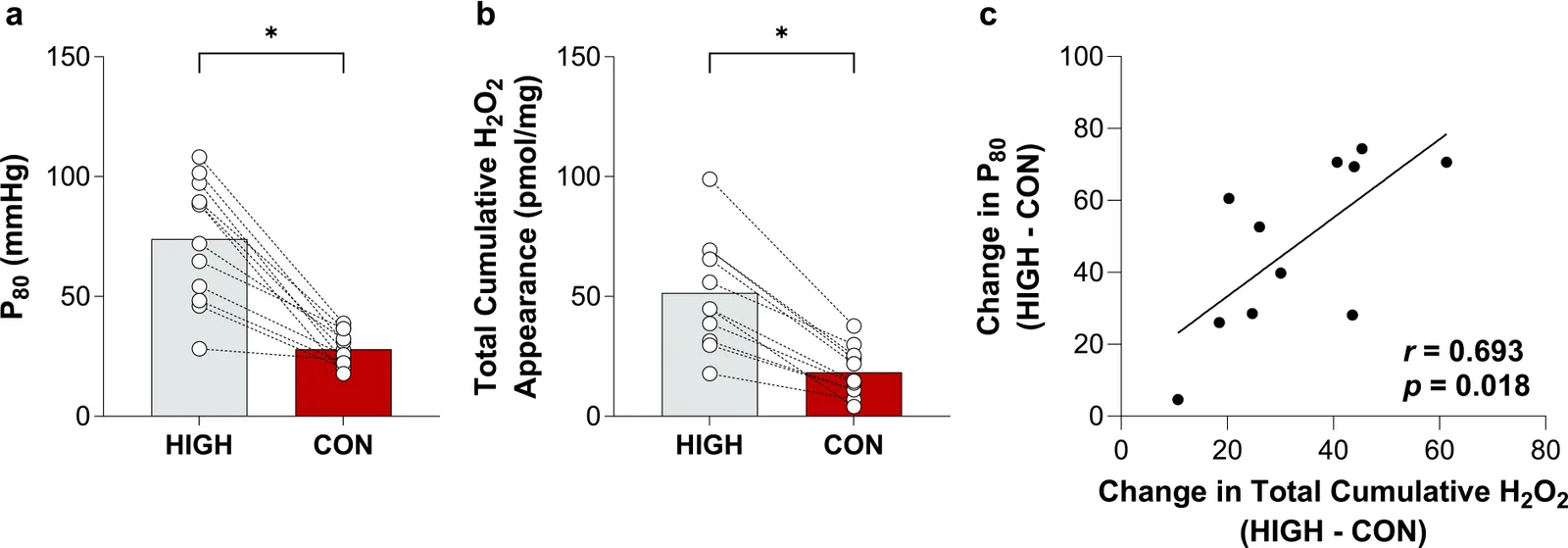
The PO_2_ at 80 % of the maximal mitochondrial respiration rate, and the total cumulative H_2_O_2_ appearance in permeabilized skeletal muscle fibers with control and high initial O_2_ concentrations. CON, control initial PO_2_ (≈127 mmHg); HIGH, high initial PO_2_ (≈327 mmHg). Panel a: PO_2_ at 80 % of V_max_ (P_80_). Panel b: Total cumulative H_2_O_2_ appearance Panel c: The relationship between the absolute change between conditions for P_80_ and total cumulative H_2_O_2_ appearance. Data are expressed as mean ± SD. n = 12 participants for panel a; *n* = 11 participants for panels b & c.

Additionally, the P_80_ was significantly greater in HIGH than CON (*p* < 0.001, Fig. 5a). The duration of the experimental protocol in HIGH was significantly longer than CON (43.4 ± 8.6 min vs. 21.3 ± 5.9 min, *p* < 0.001). Importantly, tissue weight was not significantly different between conditions (5.2 ± 1.1 mg in HIGH vs. 5.0 ± 1.1 mg in CON, *p* = 0.732).

### 3.2. Mitochondrial H_2_O_2_ appearance in CON and HIGH

All H_2_O_2_ data are for *n* = 11 because of technical issues during the protocols for one sample. The H_2_O_2_ appearance at both LEAK and V_max_ was significantly greater for HIGH than CON (*p* < 0.001 and *p* = 0.007, respectively, Fig. 4b). When normalized to respiration rate, H_2_O_2_ appearance remained significantly greater in HIGH vs CON for at both LEAK and V_max_ (*p* < 0.001 and p = 0.007, respectively, Fig. 4c). Additionally, the relative decrease in H_2_O_2_ appearance following the transition from LEAK to V_max_ was significantly greater in HIGH compared to CON (*p* < 0.001, Fig. 4d).

**Fig. 6.**
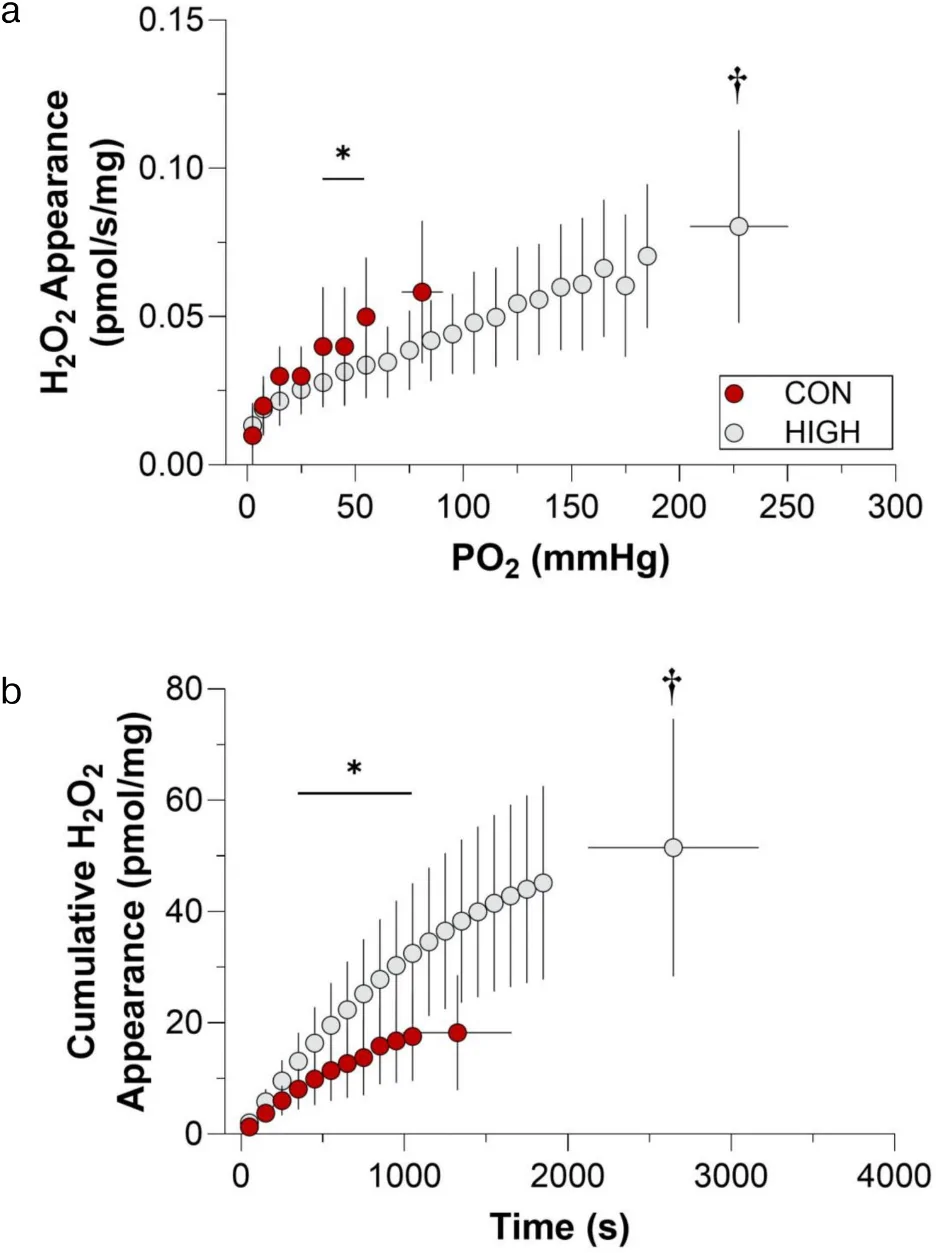
The effect of falling PO_2_ and time on mitochondrial hydrogen peroxide appearance in permeabilized skeletal muscle fibers with control and high initial O_2_ concentrations. CON, control initial PO_2_ (≈127 mmHg); HIGH, high initial PO_2_ (≈327 mmHg). Panel a: Mitochondrial hydrogen peroxide (H_2_O_2_) appearance at state 3 respiration across a falling PO_2_. Panel b: Mitochondrial cumulative H_2_O_2_ appearance at state 3 respiration over time. Data are expressed as mean ± SD. n = 11 participants. * Significant difference between CON and HIGH at a given PO_2_ or time, *p* < 0.05. † Significant difference between CON and HIGH for the peak rate of H_2_O_2_ appearance (Panel a) and the total cumulative H_2_O_2_ appearance (Panel b), *p* < 0.05.

There were significant effects for PO_2_ (*p* < 0.001), condition (*p* = 0.036), and their interaction (*p* < 0.001) for the rate of H_2_O_2_ appearance. Specifically, the rate of H_2_O_2_ appearance was significantly greater for CON than HIGH for the 35–55 mmHg PO_2_ bins (all *p* ≤ 0.028, Fig. 6a). There were also significant effects for time (*p* < 0.001), condition (*p* < 0.001), and their interaction (*p* < 0.001) for the cumulative H_2_O_2_ appearance relative to time. Specifically, these data revealed that the cumulative H_2_O_2_ appearance was significantly greater for HIGH than CON for the 350–1050 s time bins (all *p* < 0.011, Fig. 6b). The total cumulative H_2_O_2_ appearance was also significantly greater for HIGH than CON (p < 0.001, Figs. 5b & 6a). The absolute difference between conditions for the total cumulative H_2_O_2_ appearance was strongly correlated with the absolute change between conditions for the P_80_ (*r* = 0.693, *p* = 0.018, Fig. 5c).

## 4. Discussion

In the current study, we determined the impact of O_2_ availability on mitochondrial state 3 respiratory function in permeabilized human skeletal muscle fibers. To our knowledge, this is the first study to expose permeabilized muscle fiber mitochondria to CON (~123 mmHg) or HIGH (~327 mmHg) O_2_ concentrations and then measure state 3 respiration and H_2_O_2_ appearance rates until respiration ceased. In support of our first hypothesis, V_max_ was not different between CON and HIGH. As V_max_ was attained prior to a substantial increase in cumulative H_2_O_2_ appearance, no difference between conditions indicates that the higher O_2_ concentration by itself did not impair mitochondrial state 3 respiratory function. However, mitochondrial state 3 respiration rate was lower at submaximal PO_2_ values (7.55–55 mmHg) in HIGH compared to CON, supporting our second hypothesis. Together with the greater P_80_, these data indicate that mitochondrial state 3 respiration was impaired after attaining V_max_ in HIGH. Further analyses revealed that LEAK respiration and LEAK H_2_O_2_ appearance were significantly greater, while RCR was significantly less, in HIGH compared to CON, which indicate decreased efficiency and increased ROS production linked to increased non-phosphorylating respiration. Additionally, the ratio between H_2_O_2_ appearance and respiration was greater in HIGH at both LEAK and state 3 respiration, further showing decreased efficiency in hyperoxia. Finally, the total cumulative H_2_O_2_ appearance was greater in HIGH than CON, consistent with our third hypothesis, and this was strongly related to the impairment in state 3 respiration in HIGH. In conclusion, a high O_2_ concentration, by itself, does not appear to affect V_max_ in human permeabilized skeletal muscle fiber mitochondria, but the corollary increase in H_2_O_2_ exposure may diminish mitochondrial state 3 respiratory function.

### 4.1. Chamber O_2_ concentration and mitochondrial respiration

While many studies have explored the O_2_ dependence of mitochondrial respiration, most of this research was conducted using isolated mitochondria rather than permeabilized muscle fibers [20–25]. One advantage of isolated mitochondria is the negligible resistance to O_2_ diffusion, such that the generated oxygen kinetic curves accurately reflect cytochrome *c* oxidase [26]. However, an advantage of permeabilized fibers is that the in vivo cellular environment stays relatively intact [27,28]. Permeabilized muscle fibers are commonly utilized in studies that assess mitochondrial respiratory function via high-resolution respirometry [1,9,18]. Unlike isolated mitochondria, the permeabilized fibers have substantial resistance O_2_ diffusion between the respiration medium and the mitochondria [12,29]. To overcome this, studies routinely use high O_2_ concentrations (e.g., 177–355 mmHg) in the chamber to ensure that mitochondrial respiration is not constrained by O_2_ availability [9]. The fact that V_max_ was not different between conditions in the current study (Figs. 3 & 4a) supports that O_2_ availability was not initially limiting in either condition. Although the initial O_2_ concentration in CON was sufficient to elicit V_max_, this was likely not high enough to avoid O_2_ availability limitations during a typical respiration protocol [13,14], confirming that higher O_2_ concentrations are likely necessary to maintain V_max_ throughout these relatively long protocols.

### 4.2. Mitochondrial respiration and H_2_O_2_ exposure

The negligible resistance to the diffusion of O_2_ between the respiration medium and isolated mitochondria is such that the PO_2_ at cytochrome *c* oxidase is nearly equal to the PO_2_ in the respiration chamber [20,30]. This means that the hyperbolic relationship between mitochondrial respiration rate and PO_2_ can be accurately measured using chamber PO_2_, and researchers often characterize this curve using Michaelis-Menten kinetics or another regression model to indicate the PO_2_ at which respiration is limited by O_2_ availability (i.e., critical PO_2_) [31–33]. In permeabilized muscle fibers, however, the relatively greater resistance to O_2_ diffusion results in the PO_2_ at cytochrome *c* oxidase being less than the PO_2_ in the chamber and prevents the relationship between chamber PO_2_ and mitochondrial respiration rate from being hyperbolic (Fig. 2). Therefore, we opted to use a threshold PO_2_ value as an indication of an O_2_ availability limitation in mitochondrial respiration (P_80_) to avoid mathematical artifacts caused by the lack of Michaelis-Menten kinetics in permeabilized fibers. The P_80_ was 20 % greater in HIGH than CON (Fig. 5a). Interestingly, the P_80_ for HIGH does not appear to coincide with the point at which O_2_ availability limited respiration (Fig. 3). We base this on the assumption that the permeabilized muscle fiber O_2_ diffusion characteristics were similar for each condition and, therefore, a given PO_2_ in the chamber yielded similar PO_2_ values at cytochrome *c* oxidase. Thus, based on the P_80_ for CON, chamber PO_2_ values above 28 mmHg should have been enough for O_2_ availability not to limit V_max_ in HIGH (Figs. 3 & 5a). The greater P_80_ together with the lower respiration rates across submaximal chamber PO_2_ values indicate that something other than O_2_ availability impaired state 3 respiration rate in HIGH. LEAK H_2_O_2_ production, state 3 H_2_O_2_ production, and cumulative H_2_O_2_ appearance were greater in HIGH than CON (Figs. 4b, 5b, & 6b). This is consistent with two important studies that found permeabilized muscle fiber mitochondria H_2_O_2_ appearance was directly related to chamber O_2_ concentration [13,14]. With greater chamber O_2_ concentrations, more O_2_ molecules are available in the mitochondria to interact with electrons at complexes I and III to form ROS [34,35]. Although mitochondria possess endogenous antioxidant systems, such as glutathione, peroxiredoxins, and superoxide dismutase, prolonged exposure to high O_2_ may overwhelm these buffering capacities, resulting in elevated H_2_O_2_ accumulation [36]. When ROS generation outpaces the capacity of these systems, oxidative stress ensues, potentially impairing mitochondrial function and cellular homeostasis. It is well documented that ROS production is closely linked to respiratory function in skeletal muscle mitochondria [37–39] and that ROS can damage key mitochondrial proteins involved in respiration [34,40,41]. High ROS concentrations can also promote mitochondrial uncoupling that reduces efficiency and impairs respiration if oxidative damage to mitochondrial components occurs [42,43]. Consistent with this, the difference in cumulative H_2_O_2_ appearance between HIGH and CON was directly related to the difference in P_80_ in the current study (Fig. 5d). Overall, these findings support that the total exposure of skeletal muscle mitochondria to H_2_O_2_ influences state 3 respiratory function.

## 5. Potential implications

The findings of the current study have important implications for studies using permeabilized muscle fibers to assess mitochondrial respiration. Many protocols (e.g., SUIT and ADP titration) vary in duration and require relatively high chamber O_2_ concentrations to ensure O_2_ availability does not limit mitochondrial respiration. The duration and O_2_ concentration will influence the total exposure of mitochondria to H_2_O_2_ throughout the protocol, which should be considered as part of data interpretation. For the protocols in the present study, V_max_ was elicited early in the protocol before the mitochondria total H_2_O_2_ exposure substantially increased (Figs. 3 & 4a). Thus, the V_max_ was not impaired in HIGH compared to CON, but the state 3 respiration rate was impaired later in the protocols after mitochondria total H_2_O_2_ exposure increased. This may not be the case for other protocols where V_max_ occurs at a later time point. For these protocols, V_max_ may be impaired, whereas the variables earlier in the protocol may not, because of the relation to mitochondria total H_2_O_2_ exposure. Interestingly, LEAK respiration and H_2_O_2_ appearance normalized to respiration were significantly greater in HIGH, which suggests that elevated O_2_ concentrations led to decreased efficiency and increased oxidative stress [11]. H_2_O_2_ appearance per unit of O_2_ consumption was also consistently greater during LEAK compared to V_max_ in both conditions, with this difference being more pronounced in HIGH. Together, these findings suggest that hyperoxia not only increases proton conductance or electron leak, but also disproportionately enhances non-ATP-coupled H_2_O_2_ appearance, pointing to reduced coupling efficiency and elevated ROS production under high O_2_. The current data also indicate that the critical PO_2_ measured in permeabilized muscle fibers may be influenced by mitochondria total H_2_O_2_ exposure and not solely O_2_ availability. It is also important to consider that H_2_O_2_ appearance and ROS exposure will likely differ across participant cohorts and experimental conditions and, if not accounted for, may confound the mechanistic interpretation of differences in mitochondrial respiratory function. Thus, the exposure of mitochondria to ROS should be considered in studies assessing mitochondrial function ex vivo, particularly in studies where chamber O_2_ concentration varies throughout the protocol.

## 6. Experimental considerations

Several important factors should be considered when interpreting the results of this study. First, although we observed an association between elevated H_2_O_2_ exposure and impaired submaximal mitochondrial respiration, the absence of an antioxidant condition limits our ability to directly attribute this impairment to ROS. Second, the study design did not allow us to determine whether the impairment in respiration reflects a transient, reversible inhibition or irreversible mitochondrial damage. Addressing this question would require a recovery phase (i.e., reoxygenation) or sequential respiration protocols. Third, we assumed that diffusion characteristics of the samples were similar between CON and HIGH due to identical sample preparations, but prolonged exposure to elevated O_2_ and H_2_O_2_ concentrations in HIGH may have compromised mitochondrial membrane integrity as the protocol progressed. Fourth, we did not add cytochrome *c* to assess mitochondrial membrane integrity, as its addition at the start of the protocol could have interfered with subsequent respiration vs. PO_2_ measurements. Overall, the findings of this study provide compelling evidence that elevated H_2_O_2_ exposure can impair mitochondrial respiration and lay a strong foundation for future studies to determine the reversibility and mechanistic basis of this effect.

## 7. Summary and conclusion

This study is the first to comprehensively examine the impact of O_2_ concentration on mitochondrial state 3 respiration rate in permeabilized human skeletal muscle fibers, by exposing the mitochondrial to CON (~123 mmHg) or HIGH (~327 mmHg) O_2_ concentrations and then measuring respiration and H_2_O_2_ appearance rates until respiration ceased. In both conditions, V_max_ was elicited early and was not different. After V_max_ was attained in HIGH, mitochondrial state 3 respiration rates were impaired at submaximal PO_2_ values compared to CON. Mitochondrial total H_2_O_2_ exposure gradually increased throughout both protocols, and the total exposure was greater in HIGH than CON. The greater H_2_O_2_ exposure was directly related to the impairment in mitochondrial state 3 respiration in HIGH. In conclusion, a high O_2_ concentration, by itself, does not appear to affect V_max_ in the permeabilized skeletal muscle fiber preparation, but the corollary increase in H_2_O_2_ exposure may diminish mitochondrial state 3 respiratory function.

## Data Availability

Data will be made available on request.
